# Structure and Spectral Properties of Er^3+^-Doped Bismuth Telluride Near-Infrared Laser Glasses

**DOI:** 10.3390/ma17133292

**Published:** 2024-07-03

**Authors:** Fang Tan, Guoxing Xie, Yuqin Ma, Yunlong Zhang, Binhao Gao, Shunfa Cui, Dexiao Chen, Yumeng Ban, Dechun Zhou

**Affiliations:** 1 College of Science, Changchun University, Changchun 130022, China; xieguoxing97@163.com (G.X.); zyl4018@163.com (Y.Z.); gbh609511373@163.com (B.G.); csf19991119@163.com (S.C.); dexiaochen@sina.com (D.C.); 2 School of Materials Science and Engineering, Changchun University of Science and Technology, Changchun 130022, China; congyuxuan0@gmail.com (Y.M.); bym0403@126.com (Y.B.)

**Keywords:** Er^3+^-doped, bismuth tellurite glass, topological cage structure, 1.55 μm

## Abstract

TeO_2_-Bi_2_O_3_-B_2_O_3_-ZnO laser glasses doped with Er^3+^ were synthesized through an optimized melt-quenching method. The absorption spectra at 808 nm LD pumping were studied. Various spectral tests and data analyses indicate that the maximum fluorescence emission intensity can be obtained when the Er^3+^ doping concentration reaches 2%. In this case, the emission cross-section can reach up to 9.12 × 10^−21^ cm^2^ and the gain coefficient at 1.55 μm is 6.17 cm^−1^. Simultaneously, the sample has a lower phonon energy in the high-frequency band at 1077 cm^−1^, which reduces the probability of non-radiative relaxation. The calculated energy transfer coefficient C_D-A_ is 13.8 × 10^−40^ cm^6^/s, reflecting the high cross-relaxation probability of Er^3+^ in the sample, which promotes the luminescence of 1.55 μm and favors the emission in the near-infrared region. The comprehensive results demonstrate that the prepared Er^3+^-doped bismuth telluride laser glass can be used as a promising and high-quality gain material for near-infrared lasers.

## 1. Introduction

It is well-known that Er^3+^ emits luminescence when effectively excited by laser diode pumping. This luminescence occurs with a jump at the ^4^I_13/2_→^4^I_15/2_ energy level. The center of the spectrum is located at 1550 nm. Rare-earth-ion-doped laser materials have achieved much attraction due to their extensive applications, including display devices, medical imaging, fiber-optic sensing, photonic crystal fibers, fiber-optic amplifiers (EDFAs), optical temperature sensors (OTSs), solid-state lasers, etc. [1,2,3,4,5,6]. Although it has long been of wide interest to researchers, it remains a hot topic to this day. As research has progressed, the types of glass matrices selected have become more varied. The main purpose of these matrices has changed: more expansion is obtained in improving the intensity as well as the efficiency of the Er^3+^ radiative excursion. Various glass matrices influence the electron layer structure of rare earth ions. Thus, discovering excellent matrix glass is also an essential aspect in achieving near-infrared intensity enhancement. Multi-component glass fibers present several benefits when compared to SiO_2_ glass. These benefits include lower phonon energy, good thermal stability, larger full-width at half-maximum (FWHM) values, and greater absorption/emission cross-sections. Examples of such glasses include fluoride glass, tellurite glass, and germanate glass. Furthermore, matrix glasses that contain heavy oxides, including TeO_2_, Bi_2_O_3_, and others, show promise as material species for near-infrared, fiber-optic sensing, and non-linear optic fields [7,8,9]. Tellurite glass is a suitable matrix glass material for near-infrared laser materials due to its many network structural units, adjustable glass compositional interval, low phonon energies (700 cm^−1^), good thermal stability, high refractive indices (~1.8–2.2), and ability to accommodate high rare earth doping concentrations and higher laser damage thresholds [10].

Moreover, it is crucial to include glass-forming agents, and boron oxide (B_2_O_3_) is commonly preferred. B_2_O_3_ glasses offer significant advantages over other types of glasses, including high mechanical properties, chemical and thermal stability, and a low melting point [11]. However, the network structure generates high phonon energies, which increase the non-radiative emission of tellurite glass. An appropriate amount of heavy metal oxides can be added to reduce the phonon energy and increase the energy transfer efficiency [1]. Recent research findings have indicated that adding ZnO to tellurite glass can increase its density, while reducing both the softening point and glass transition temperature of the binary glass [12,13]. At present, the majority of studies concentrate on reporting the luminescence properties of heavy metal oxide glass matrices that contain rare earth ion-doped compounds such as ZnO, Bi_2_O_3_, and so on. However, the weak luminescence of Te-activated glass in the bands used for near-infrared communication (e.g., O-L band) means that there has been less exploration into Te-activated glass components, and the combination of Er^3+^-doped tellurite glass with ZnO, B_2_O_3_, and Bi_2_O_3_ oxides remains a topic that has not yet received extensive attention.

In this work, Er^3+^-doped bismuth tellurite glasses with the composition TeO_2_-Bi_2_O_3_-B_2_O_3_-ZnO was prepared by conventional melt-quenching method. The thermal stability of tellurite glass and the luminescence properties of rare earth ions were investigated experimentally and theoretically. The Judd–Ofelt intensity parameters, spontaneous leapfrog chances, fluorescence branching ratios, and radiative lifetimes were calculated. The maximum absorption cross-section recorded was 3.0 × 10^−21^ cm^2^, while the emission cross-section was 9.12 × 10^−21^ cm^2^. Subsequently, gain coefficients were calculated. Finally, the energy transfer mechanism of Er^3+^ was analyzed.

## 2. Materials and Methods

Er^3+^-doped tellurite glasses with the molar composition of 80TeO_2_-5Bi_2_O_3_-5B_2_O_3_-10ZnO-xEr_2_O_3_ (where x = 1, 1.5, 2, 2.5, 3 mol%, which were labeled as TBBZ1 to TBBZ5) were prepared by the melt quenching method with raw materials including TeO_2,_ B_2_O_3_, ZnO, and Er_2_O_3_, all of which have a purity of 99.99%. A precisely measured 10 g of the raw material was mixed well, ground thoroughly in an onyx mortar, melted in an alumina oxide crucible at 1000 °C for about 30 min under the protection of an O_2_/N_2_ atmosphere. The glass samples were annealed at 370 °C for 3 h in order to eliminate thermal stress, and then slowly reduced to room temperature at a cooling rate of 10 °C/h. Finally, the glass samples were cut and polished to a thickness of 2 mm for subsequent optical and spectral measurements.

Distilled water was used as the impregnation solution and the density of the TBBZ3 glass was measured to be 4.7841 g/cm^3^ using the Archimedes’ method. Refractive index at 1550 nm measured with SPA 4000TM prism coupler at room temperature was 1.81. The glass transition temperature (T_g_) and crystallization temperature (T_x_) were measured by differential scanning calorimeter (DSC) at a heating rate of 10 °C/min under an N_2_ atmosphere. In addition, the absorption spectra were tested by a PerkinElmer Lambda 900 UV/VIS/NIR spectrophotometer and the emission spectra were measured with a Triax 320 type spectrometer (Jobin Yvon Corp., Stow, MA, USA) under 808 nm laser diode excitation. Raman and infrared spectra were measured using a Renishaw inVia Raman microscope and a Spectrum Two type spectrometer (PerkinElmer, Waltham, MA, USA).

## 3. Results

### 3.1. Thermal Stability

**Figure 1:**
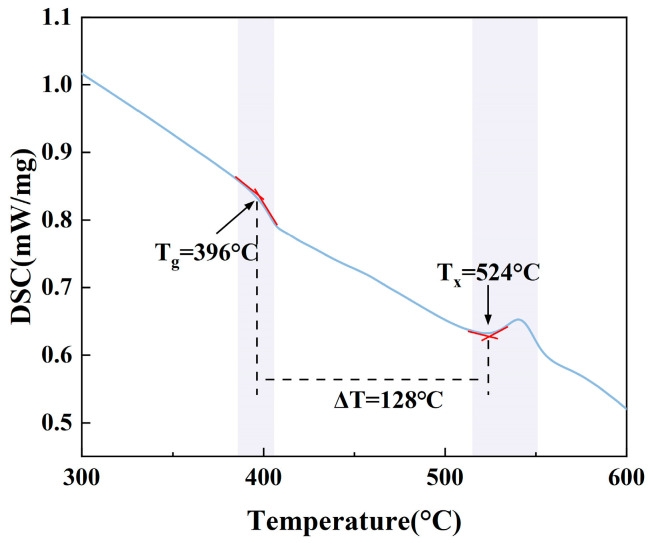
DSC curve of TBBZ host glass.

Thermal stability has an impact on fiber formation performance, the glass transition temperature (T_g_), the crystallization temperature (T_x_), and their difference ΔT = T_x_ − T_g_ is an important evaluation index in the thermal stability analysis of glasses. Stretching of fiber-optic cables is preferred when the temperature difference (ΔT) exceeds 100 °C [14]. Figure 1 shows the DSC curves of TBBZ glass with values of T_g_, T_x_, and ΔT of 396 °C, 524 °C, and 128 °C, respectively. Its ΔT is larger than that of other Er^3+^-doped tellurite glass samples (TeO_2_-ZnO-Na_2_O, ΔT = 127.2 °C) [15], germanate glass (Bi_2_O_3_-GeO_2_-Ga_2_O_3_-Na_2_O, ΔT = 121 °C) [16], and germanium tellurite glass (GeO_2_-TeO_2_-Na_2_O, ΔT = 106 °C) [17]. The further improvement in thermal stability may be attributed to the new evolution of the original glass network structure due to the introduction of B_2_O_3_. Due to the reduced atomic radius of boron, the repulsion between non-bonding electrons is somewhat diminished, facilitating a tighter bonding between atoms. This, together with TeO_2_, modulates the population of complex oxygen ions, thereby enhancing the thermal stability of the poly glasses presented in this paper [18]. As a result, the tellurite glass produced in this study shows favorable thermal stability, which is advantageous for the formation of fibers. 

### 3.2. XRD and Transmission Spectroscopy

**Figure 2:**
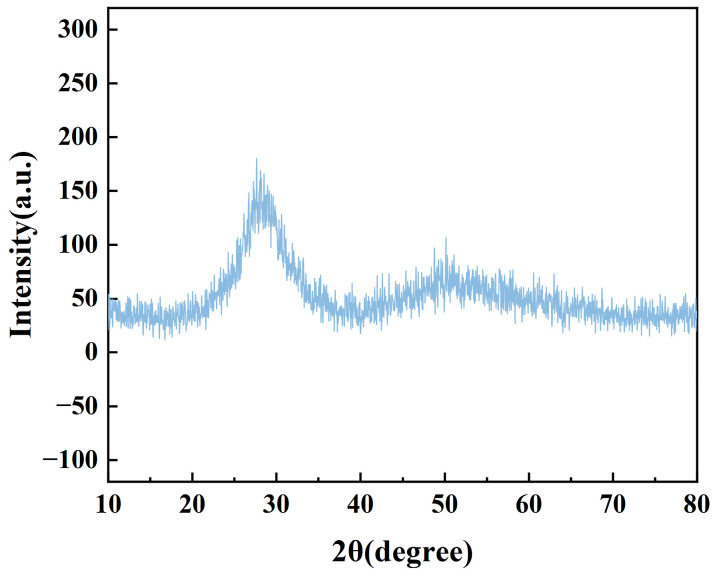
The XRD pattern of TBBZ glass sample.

To investigate the structure of tellurite glass, the sample material was characterized and analyzed. The XRD spectra of the TBBZ glass samples are given in Figure 2. As can be seen from the figure, there are two broad bands at 2θ equal to 30°, as well as near 50° without sharp crystal peaks, indicating that the prepared glass is completely amorphous in nature and does not give rise to the phenomenon of precipitation of crystals, presenting an amorphous state.

**Figure 3:**
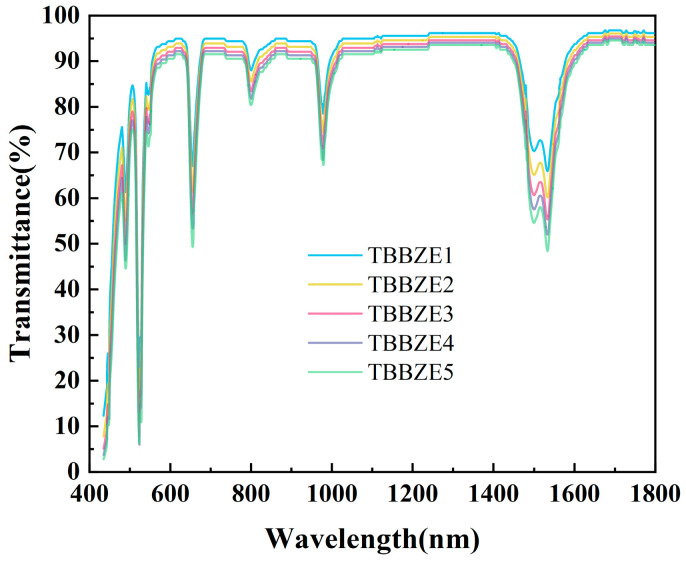
Transmission spectra of TBBZ1-5 glass sample.

In addition, the Er^3+^-doped TeO_2_-Bi_2_O_3_-B_2_O_3_-ZnO glass has a high transmittance, as shown in Figure 3. The transmittance of the glass in the near-infrared band can reach 90% with high transmittance, which is an important reference value for improving the optical properties of tellurite glass. The overall transmittance decreases slightly with increasing Er^3+^-doping concentration, which is due to absorption by the Er^3+^ ions. The spectral positions and shapes of the glasses at different rare earth ion doping concentrations are approximately the same, approximately due to the fact that the Er^3+^ ions do not aggregate in the local ligand field but are uniformly distributed in the tellurite glass network, which is similar to previous reports [19].

### 3.3. Raman Spectrum and FT-IR Spectra

**Figure 4:**
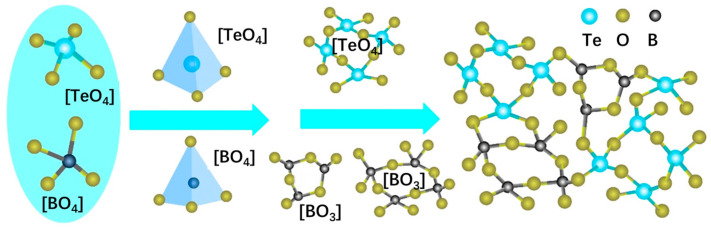
Schematic diagram of topological cage structure evolution of TBBZ glass network.

In order to investigate the physical structure of TBBZ glass, an analytical discussion of the glass network was performed. Typically, Te^4+^ ions have a larger radius and generate a looser glass network. Whereas B^3+^ ions has a smaller radius, the incorporation of B^3+^ ions results in the formation of BO_3_ triangles and BO_4_ tetrahedra within the glass. These groups combine to form a variety of three-dimensional topological cages comprising borate or diborate rings, metaborate rings, and others (see Figure 4). Such a structure can assist in modulating the degree of polymerization of the tellurite glass topological cage structure, promoting the NIR luminescent centers to be properly spaced from each other and suppressing non-radiative relaxation; the network modifier (ZnO) provides free oxygen in the glass structure and induces the transformation of the borate structural unit from BO_3_ to BO_4_, which improves the polymerization of the glass network and further promotes the formation of the [TeO_4_] topological cage. Figure 4 demonstrates the involvement of boron oxide in the modulation of the topological cage structure of the Er^3+^-doped tellurite glass network in this paper. Erbium and bismuth, as activators, are located outside the topological cage and have an insignificant influence on the composition of the glass network.

**Figure 5:**
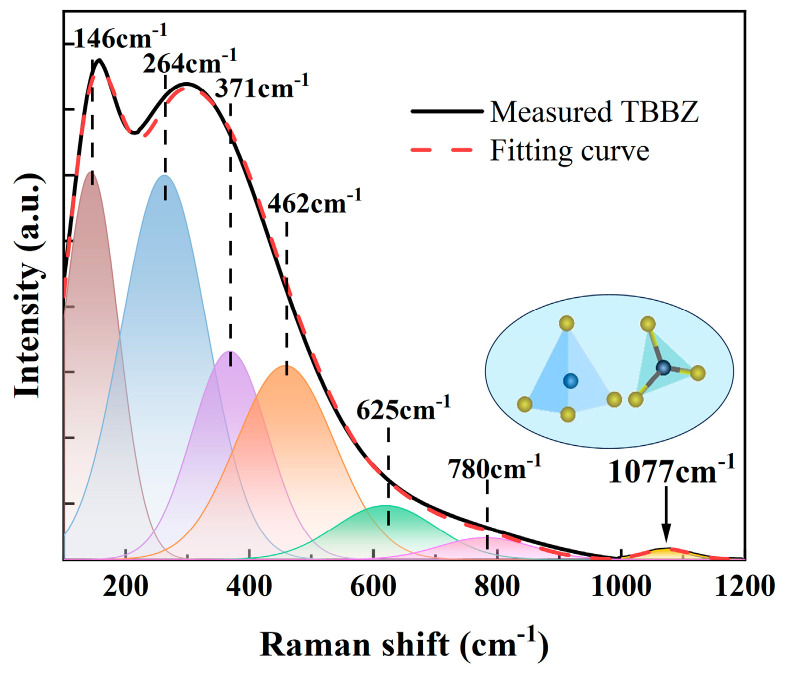
The Raman spectra of TBBZ host glass.

Figure 5 shows the Raman spectra of the matrix glass measured using the Gaussian fitting method. There are seven distinct spectral bands near 146 cm^−1^, 264 cm^−1^, 371 cm^−1^, 462 cm^−1^, 625 cm^−1^, 780 cm^−1^, and 1077 cm^−1^. Among them, the Gaussian peak at 146 cm^−1^ is due to the Bi^3+^ vibration in [BiO_6_] [20], and the Gaussian peaks at 264 cm^−1^, 371 cm^−1^, and 462 cm^−1^ are due to the bending vibration or symmetric stretching of Te-O-Te in the [TeO_4_] (tbps), [TeO_3+δ_], and [TeO_3_] (tps) moieties [21] and the [BiO_3_], and Bi-O-Bi symmetric stretching [22] in [BiO_6_] is caused by the joint action. Unlike the peak around 650 cm^−1^ in normal tellurite glasses, the introduction of B_2_O_3_ shifts the peak there to 780 cm^−1^, probably because the addition of B_2_O_3_ breaks the Te-O-Te bond, resulting in the transformation of [TeO_4_] into [TeO_3_] and [TeO_3+δ_] units [22]. With the addition of B_2_O_3_, the formation of a new peak at 1077 cm^−1^ is associated with the B-O and B-O-B symmetric stretching vibrations in [BO_3_] and [BO_4_] [23]. Therefore, the maximum phonon energy of the matrix glass is 1077 cm^−1^ and the low phonon energy reduces the probability of non-radiative relaxation and favors Er^3+^ emission in the NIR region.

**Figure 6:**
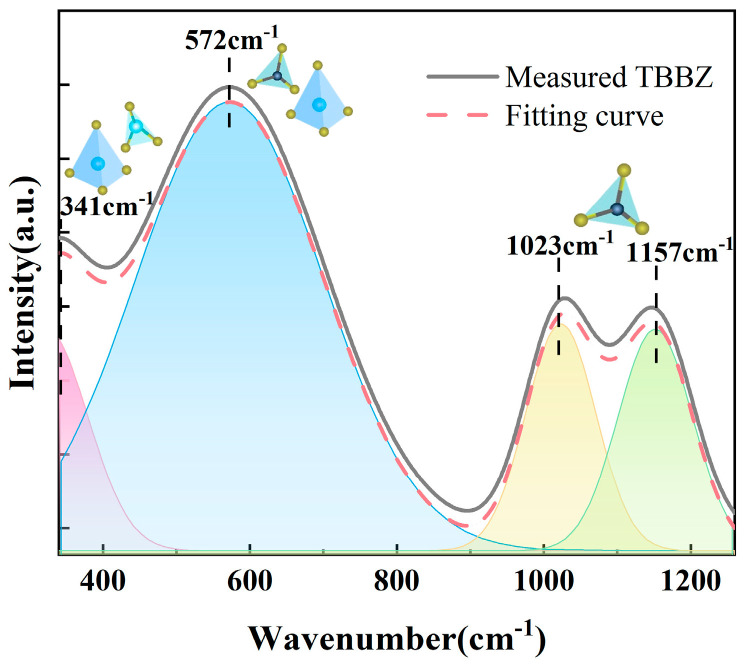
FT-IR spectra of TBBZ host glass.

In addition, a Gaussian decomposition of the infrared spectral lines of the TBBZ glass was performed in order to determine the molecular vibrations in the TBBZ glass network. The infrared spectrogram at 330–1200 cm^−1^ is given in Figure 6. Four distinct spectral bands are present at approximately 341 cm^−1^, 572 cm^−1^, 1023 cm^−1^, and 1157 cm^−1^. The spectral band at 341 cm^−1^ corresponds to the symmetric stretching or bending vibrations of Te-O-Te in [TeO_4_] (tbps), [TeO_3+1_], and [TeO_3_] (tps) [24]. The 572 cm^−1^ peak could potentially originate from the bending vibration of B-O-B in [BO_3_] and the stretching vibration of Te-O in [TeO_4_] [25]. The bands at 1023 cm^−1^ and 1157 cm^−1^ are attributable to B-O antisymmetric stretching vibrations in [BO_3_] [26,27].

### 3.4. Absorption Spectrum and Judd–Ofelt Analysis

**Figure 7:**
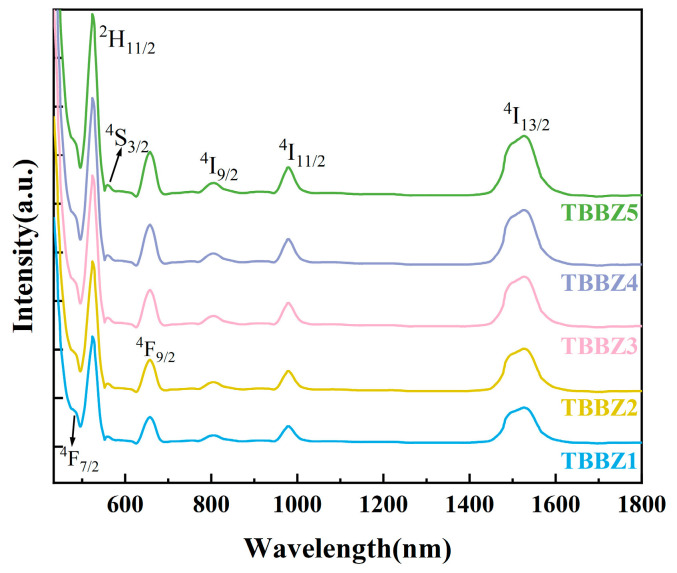
The absorption spectra of Er^3+^-doped tellurite glasses.

The absorption spectra of Er^3+^-doped tellurite glass samples are shown in Figure 7 from 400 to 2000 nm. The intensity of the absorption peaks of the glass samples was significantly enhanced when the Er^3+^ doping concentration was increased. Observation of the Er^3+^ absorption spectrum revealed the presence of seven absorption peaks on the spectral line, and the absorption bands located at 489, 529, 552, 655, 798, 975, and 1533 nm corresponded to the electronic leaps from the ground state (^4^I_15/2_) to the excited states ^4^F_7/2_, ^2^H_11/2_, ^4^S_3/2_, ^4^F_9/2_, ^4^I_9/2_, ^4^I_11/2_, and ^4^I_13/2_, respectively. The absorption band of Er^3+^ at less than 300 nm could not be measured due to the rapid increase in the electronic absorption fringes [28]. This indicates the uniform binding of Er^3+^ into the tellurite glass network. Er^3+^ exhibits a clear absorption band at approximately 808 nm (^4^I_15/2_→^4^I_9/2_), indicating the potential for commercial 808 nm laser diodes to excite singly doped Er^3+^ in tellurite glass fibers.

To examine the luminescent properties of tellurite glasses, the Judd–Ofelt theory [29,30] was used to analyze both the local structure and the luminescent properties of trivalent rare earth ions. Based on the absorption spectra, the J-O intensity parameter Ω_λ(λ=2,4,6)_ of the TBBZ glass can be calculated by the least squares method. Table 1 displays the J-O parameters of various tellurite matrix glasses doped with different concentrations of Er^3+^. The parameters of J-O intensity (Ω_2_, Ω_4_, Ω_6_) exhibit a declining trend as the concentration of Er^3+^ doping increases. It should be noted that the calculated glass strength parameters Ω_2_ > Ω_4_ > Ω_6_ are similar to the phenomenon previously found in tellurite glasses [31]. In general, Ω_2_ correlates with the ligand symmetry of the immediate surroundings and is highly responsive to the covalent bonding between rare earth ions and anionic ligands. Larger values of Ω_2_ indicate lower ligand symmetry and a more compact glass network structure. The Ω_2_ values of TBBZ glass in Table 1 exceed those of other matrix glasses; this could be attributed to the disruption of the glass network through doping with oxides such as B_2_O_3_ and ZnO, leading to the improved stability of the TBBZ glass. While the value of Ω_2_ decreases with continuous Er^3+^ doping, the reason for this phenomenon may be the conversion of [TeO_3_](tp) to [TeO_4_](tbp) units and the subsequent decrease in ligand bond covalency of Er^3+^ [32]. Ω_4_ is related to the viscosity of the glass and decreases with the increasing Er^3+^ doping concentration. Ω_6_ is proportional to the body glass stiffness, and as Ω_6_ increases, its corresponding mechanical properties decrease. Meanwhile, a reduction in the covalency between rare earth ions and oxide ions appears to markedly decrease the value of Ω_6_ [32].

After obtaining the intensity parameter Ω_λ(λ=2,4,6)_, it was used to calculate the glass TBBZ3’s spontaneous leap chance A_rad_, fluorescence branching ratio β, and radiation lifetime τ. As shown in Table 2, the spontaneous jump chance of Er^3+^ in TBBZ3 at ^4^I_11/2_→^4^I_13/2_ is about ~25 s^−1^, which is higher than that of fluorotelluride [36] (21 s^−1^), ZBLAN (18 s^−1^), and fluoroaluminate [37] (19 s^−1^). The higher spontaneous transition probability Arad is easier to achieve laser emission and can be effectively used in the field of 2.7 μm lasers [38]. The radiative lifetime at ^4^I_13/2_→^4^I_15/2_ is 3.52 ms, which is higher than the reported TeO_2_-ZnO-ZnF_2_ tellurite glass [39] (3.23 ms) and bismuthate glass [40] (2.94 ms). The above studies show that the prepared TBBZ3 glass has a strong potential for development in the application of lasers in the near-infrared band.

### 3.5. Fluorescence Spectrum and Lifetime

**Figure 8:**
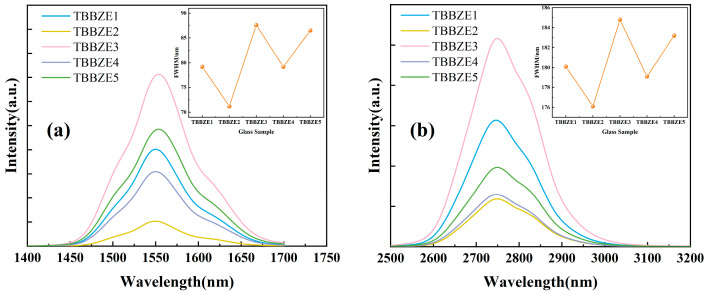
(**a**) The 1.55 µm fluorescence spectra of TBBZ1-5 glasses; (**b**) 2.7 µm fluorescence spectra of TBBZ1-5 glasses.

The topological cage structure demonstrated in Figure 4 can effectively regulate the ligand field around rare earth ions and thus the luminescence behavior. Figure 8a,b shows the fluorescence spectra of TBBZ1-5 in the range of 1400–1700 nm and 2500–3200 nm, respectively, both produced by 808 nm LD pump excitation. The emission peak at the central wavelength of 1550 nm in Figure 8a is due to Er^3+^ during the energy transfer from ^4^I_13/2_ to ^4^I_15/2_. The emission peak at the central wavelength of 2750 nm in Figure 8b is caused by the jumping of Er^3+^ at the ^4^I_11/2_→^4^I_13/2_ energy level. From the observation, it can be seen that the fluorescence intensity of TBBZ glass at both 1550 nm and 2750 nm shows a repeated decreasing and increasing trend as the Er^3+^ concentration is doped from 1 mol% to 3 mol%, and the fluorescence intensity reaches the peak at 2 mol% concentration. The phenomenon in which a reduction in intensity occurs is known as fluorescence concentration quenching, which may be due to aggregation between Er^3+^ ions caused by an increase in Er_2_O_3_ content, which leads to enhanced interactions and increased probability of radiationless leaps, resulting in a decrease in the luminescence efficiency, and a similar trend has been found in tellurite glasses [31]. The insets in Figure 8a,b show the full-width values of the fluorescence half-peaks for each Er^3+^ doping concentration at 1.55 μm and 2.7 μm, respectively. The effective fluorescence half-peak full-width (Δ*λ*_eff_) can be calculated from the following equation:

(1) $$\Delta \lambda_{\mathrm{eff}}=\frac{\int I\left(\lambda \right)\;d\lambda }{I_{\max}}$$

where *I*(*λ*) is the fluorescence intensity at the wavelength *λ* and *I*_max_ is the maximum intensity of the fluorescence emission. From the figure, it can be seen that increasing the Er^3+^ doping concentration in the range of the 1.55 μm and 2.7 μm bands shows a repeated decreasing and increasing trend in the FWHM in line with the change in fluorescence intensity. Both peak at a doping concentration of 2%, 87.56 nm compared to 184.8 nm. The larger half-peak full-width value is crucial for optimizing the bandwidth characteristics of the glass.

**Figure 9:**
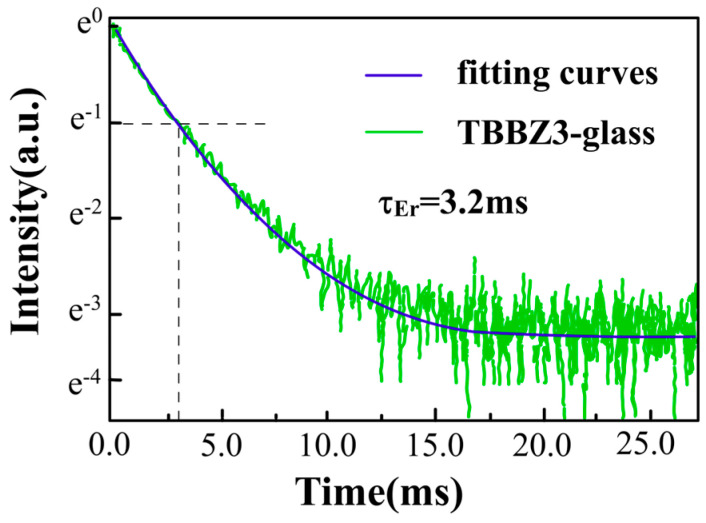
Fluorescence decay curves of the Er^3+^:^4^I_13/2_ level in TBBZ3 glass.

The fluorescence decay of Er^3+^ also provides important information on luminescence and energy transfer processes, Figure 9 shows the fluorescence decay curves at 1.55 μm for the Er^3+^:^4^I_13/2_ energy level in the TBBZ3 glass sample under pulsed excitation at 808 nm LD, with the lifetimes averaged. It is well-known that the energy level lifetime of rare earth ions refers to the length of time experienced when the fluorescence intensity decays to the highest intensity of e^−1^. Fitting by exponential decay function, the fluorescence lifetime (*τ*_mea_) of TBBZ3 glass was measured to be 3.2 ms, which is much longer than that of bismuthate glass (2.05 ms) [9], zinc fluoride glass (2.05 ms) [41], and fluorotelluride glass (1.36 ms) [42]. Increased longevity is advantageous for the population accumulation towards achieving lasers.

The quantum efficiency *η* of the ^4^I_13/2_ energy level of Er^3+^ can be calculated by reference to Equation (2):

(2) $$\eta =\frac{\tau_{\mathrm{mea}}}{\tau_{\mathrm{rad}}}\times 100\%$$

where *τ*_mea_ is the measured fluorescence lifetime and *τ*_rad_ is the calculated radiation lifetime. The quantum efficiency of the Er^3+^:^4^I_13/2_ energy level in TBBZ3 is 90%, which is higher than that of the Er^3+^-doped fluoride glass (21.29%) [19], the high quantum efficiency guarantees ample gain for the 1.55 μm laser, which is a promising contender for 1.55 μm laser realization.

### 3.6. Absorption and Emission Cross-Sections and Gain Coefficients

To characterize the spectral properties of TBBZ glass in the NIR region, the absorption and emission cross-sections at 1.55 μm were calculated and analyzed. The absorption cross-section *σ*_abs_ can be calculated by the Beer–Lambert law [43]:

(3) $$\sigma_{\mathrm{abs}}\left(\lambda \right)=\frac{2.303\mathrm{lg}\left(I_{0}/I\right)}{N_{0}L}$$

where lg(*I*_0_/*I*) is the absorption intensity; *I*_0_ is the incident light intensity; *I* is the transmitted light intensity; *N*_0_ is the rare earth ion concentration; and *L* is the sample thickness.

**Figure 10:**
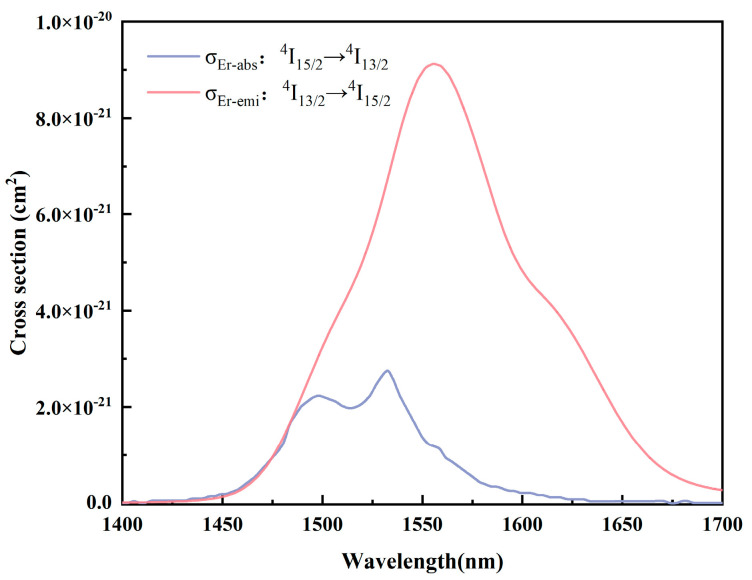
Absorption and emission cross-sections of Er^3+^:^4^I_13/2_↔^4^I_15/2_ transition in TBBZ3 glass.

The emission cross-section *σ*_emi_ can be calculated by the Füchtbauer–Ladenburg [14] equation:

(4) $$\sigma_{\mathrm{emi}}=\frac{\lambda^{4}A_{\mathrm{rad}}}{8n^{2}c}\times \frac{\lambda I\left(\lambda \right)}{\int \lambda I\left(\lambda \right)d\lambda }$$

where *λ* is the wavelength; *A*_rad_ is the spontaneous jump chance; *c* is the speed of light; *n* is the refractive index; and *I*(*λ*) is the fluorescence intensity at wavelength *λ*. Figure 10 shows the absorption and emission cross-sections produced by Er^3+^ during energy transfer at two energy levels, ^4^I_13/2_ and ^4^I_15/2_, with peaks *σ*_abs_ = 4.49 × 10^−21^ cm^2^ and *σ*_emi_ = 9.12 × 10^−21^ cm^2^ at 1532 nm and 1556 nm, respectively. This is higher than the previously reported Er^3+^-doped tellurite glass [39] (8.7 × 10^−21^ cm^2^) and fluoroaluminate glass [44] (5.30 × 10^−21^ cm^2^). The high absorption–emission cross-section is conducive to the enhancement of the pump absorption efficiency and the effective emission of Er^3+^ at 1.55 μm, which further proves that the TBBZ3 glass is an excellent dielectric material for applications in the near- and mid-infrared.

**Figure 11:**
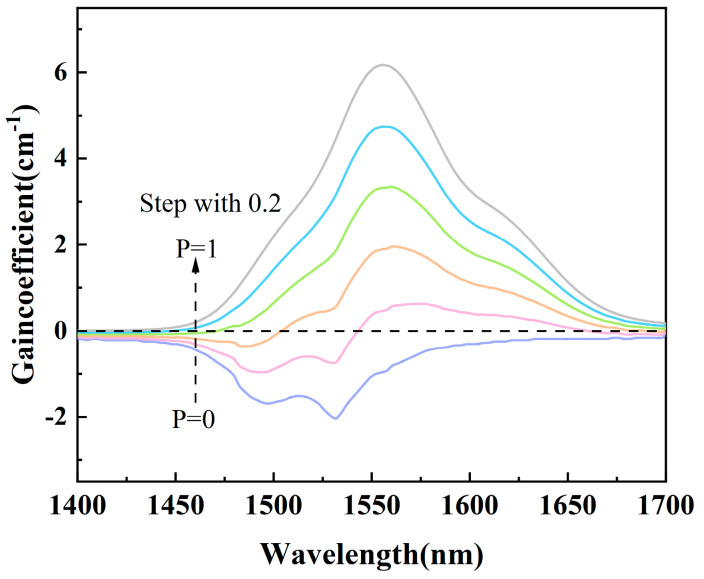
Gain coefficient spectra of TBBZ3 glass.

The gain coefficient *G*(*λ*) [44] can be approximated based on the previously calculated absorption *σ*_abs_ and emission cross-section *σ*_emi_:

(5) $$G\left(\lambda \right)=N\left[P\sigma_{\mathrm{emi}}\left(\lambda \right)-\left(1-P\right)\sigma_{\mathrm{abs}}\left(\lambda \right)\right]$$

where *N* denotes the concentration of rare earth ions and *P* is the population inversion factor. Figure 11 gives the gain coefficients calculated for the TBBZ3 glass at 1.55 μm from the range 0–1 in steps of 0.2. Positive gain at 1550 nm is achieved when the inversion factor *P* reaches 0.4 or higher, and the low inversion factor indicates that the Er^3+^-doped tellurite glass has a low pumping threshold at 1.55 μm. The gain coefficient with inversion factor *P* = 1 peaks near 1556 nm with a maximum gain coefficient *G*_max_ = 6.17 cm^−1^, which is superior to fluoride glass [44] (1.15 cm^−1^), as well as other tellurite glasses [45] (0.81 cm^−1^). Due to its high gain performance, TBBZ3 glass has the potential to be used as a gain medium material in the field of rare-earth-doped fiber lasers within the 1.55 μm band.

### 3.7. Energy Transfer Mechanism and Microscopic Parameter

**Figure 12:**
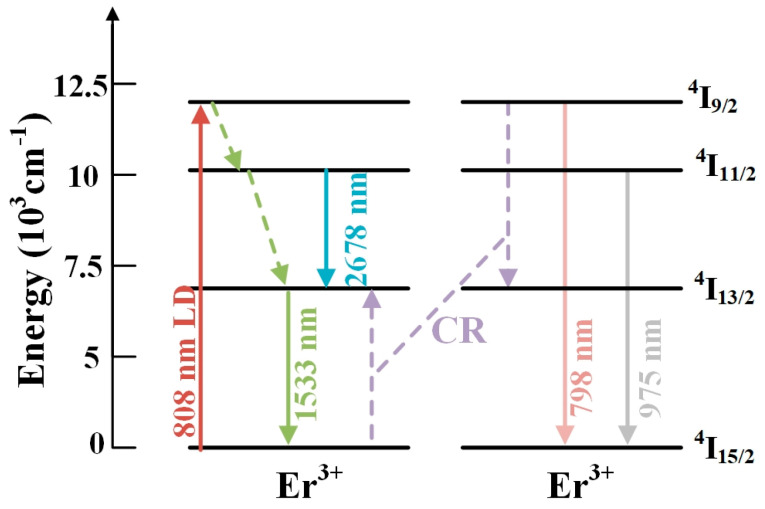
Energy level transition diagram of Er^3+^.

Figure 12 shows the energy level and energy transfer process diagram of Er^3+^ in TBBZ glass during 808 nm LD pump excitation. The energy of Er^3+^ at ^4^I_15/2_ is excited to ^4^I_9/2_ by ground state absorption (GSA). A portion of the Er^3+^ energy is transferred to the neighboring ^4^I_9/2_ energy level by means of energy transfer (EM). Due to the narrow energy gap between ^4^I_9/2_ and ^4^I_11/2_, Er^3+^ emits at 2678 nm from the ^4^I_9/2_ level to ^4^I_11/2_ by means of non-radiative transitions to ^4^I_13/2_ and subsequently to ^4^I_13/2_, and emits at 1533 nm when it returns from the ^4^I_13/2_ level to ^4^I_15/2_. The fluorescence intensity of TBBZ glass at 1.53 μm rises with increasing Er^3+^ doping concentration. This increase can be attributed to the cross-chattering process (CR) between ^4^I_9/2_→^4^I_13/2_ and ^4^I_15/2_→^4^I_13/2_.

To further investigate the energy transfer process, Dexter’s theory was used to calculate the microscopic parameters of the energy transfer between Er^3+^ based on the absorption and emission cross-sections of rare earth ions. The microscopic energy transfer probability between rare earth ions is expressed by the following equation [14]:

(6) $$W_{D\text{-}A}=\frac{C_{D\text{-}A}}{R^{6}}$$

where *D* and *A* denote donor and acceptor ions, respectively, and *R* is the distance between the donor and acceptor. The energy transfer constant is defined as follows [14]:

(7) $$C_{D\text{-}A}=\frac{R_{C}^{6}}{\tau_{D}}$$

In this equation, *R_C_* is the critical radius of the interaction and *τ_D_* is the intrinsic lifetime. When examining phonon involvement, the constant for energy transfer can be determined using the subsequent equation [14]:

(8) $$C_{D\text{-}A}=\frac{6cg_{\mathrm{low}}^{D}}{{\left(2\pi \right)}^{4}n^{2}g_{\mathrm{up}}^{D}}\sum_{0}^{\infty }e^{-\left(2\bar{n}+1\right)S_{0}}\frac{S_{0}^{m}}{m!}{\left(\bar{n}+1\right)}^{m}\int \sigma_{\mathrm{emis}}^{D}\left(\lambda_{m}^{+}\right)\sigma_{\mathrm{abs}}^{A}\left(\lambda \right)d\lambda$$

where *c* is the speed of light; *n* is the refractive index of the glass; and $g_{\mathrm{low}}^{D}$ and $g_{\mathrm{up}}^{D}$ are the simplicity of the lower and upper energy levels of the donor, respectively. *ħω*_0_ is the maximum phonon energy (1077 cm^−1^ in this paper), *n* = (1/e^*ħω*0/*KT*^ − 1) is the average phonon mode occupancy at temperature *T*, *m* is the number of phonons involved in the energy transfer, *S*_0_ is the Huang Kun factor, which has a value of 0.31 for Er^3+^ ions, and $\lambda_{m}^{+}$ = (1/*λ* − *mħω*_0_) is the *m* phonon emission corresponding to the wavelength.

**Figure 13:**
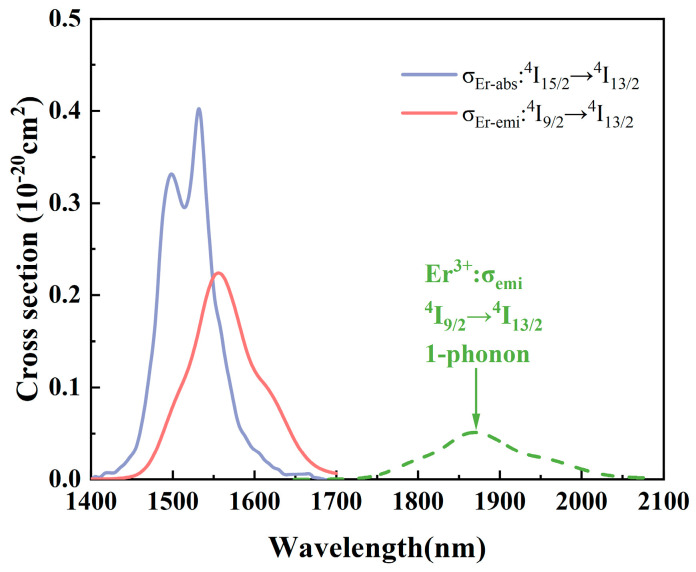
Emission and absorption cross-section of Er^3+^ at ^4^I_9/2_ + ^4^I_15/2_→^4^I_13/2_ + ^4^I_13/2_ level and edge band diagram of m phonon (m = 1) emission in ^4^I_9/2_→^4^I_13/2_ transition.

The energy transfer coefficients were analyzed by finding the TBBZ3 glass cross-retardation (CR) probability according to Equations (7) and (8). Figure 13 illustrates the emission and absorption cross-sections of Er^3+^:^4^I_9/2_ + ^4^I_15/2_→^4^I_13/2_ + ^4^I_13/2_, along with the m-phonon (m = 1) emission sidebands of the donor during the Er^3+^:^4^I_9/2_→^4^I_13/2_ transition. From the figure, it can be seen that the CR process of the prepared samples is a multi-phonon mechanism, with 0-phonon-assisted accounting for the majority (99.999%); this is a resonance process. Table 3 shows the phonon number, probability share, and microscopic parameters of the CR process in TBBZ3 glass. The process has an energy transfer coefficient C_D-A_ of 13.8 × 10^−40^ cm^6^/s and a favorable cross-chattering process, which enhances the luminescence of 1.55 μm.

## 4. Discussion

Er^3+^-doped TeO_2_-Bi_2_O_3_-B_2_O_3_-ZnO glasses were prepared by melt quenching and characterized for their thermal stability, matrix structure, and spectral properties. The results show that the Er^3+^-doped bismuth tellurite glass has good thermal stability (ΔT of 128 °C) and low phonon energy (1077 cm^−1^). The intensity parameters Ω_2,4,6_ and other spectral parameters were obtained by Judd–Ofelt theoretical calculations. In this paper, TBBZ3 (Er^3+^ = 2%) glass was prepared to achieve a high spontaneous leaping chance *A*_rad_ (25 s^−1^) at ^4^I_11/2_→^4^I_13/2_, as well as a high radiation lifetime *τ* (3.52 ms) at ^4^I_13/2_→^4^I_15/2_. The high-frequency band at 1077 cm^−1^ leads to a topological transition, giving rise to a deep topological cage structure of [BO_3_], [BO_4_]. In addition, the TBBZ3 glass achieved the highest fluorescence emission intensity and bandwidth values when pumped at 808 nm, with an emission cross-section (*σ*_emi_) of 9.12 × 10^−21^ cm^2^. It also exhibited satisfactory gain performance at 1.55 μm (*G*_max_ of 6.17 cm^−1^). Taken together, it shows that the Er^3+^-mono-doped TeO_2_-Bi_2_O_3_-B_2_O_3_-ZnO glass has good prospects for applications in laser materials in the near-infrared region, which can be further explored in the future with the goal of lowering the phonon energy and reducing the probability of non-radiative leaps.

## 5. Conclusions

In this paper, Er^3+^-doped TeO_2_-Bi_2_O_3_-B_2_O_3_-ZnO glasses were successfully prepared by melt quenching method. Testing revealed that the rare-earth-(Er^3+^)-doped bismuth tellurite glasses have more stable thermodynamic properties and lower maximum phonon energies. The calculated Ω_2,4,6_ using J-O theory indicates that the samples have superior optical properties, a higher spontaneous radiation probability, and a longer radiation lifetime (^4^I_11/2_→^4^I_13/2_). Furthermore, the sample exhibits a broad fluorescence emission bandwidth and an emission cross-section (9.12 × 10^−21^ cm^2^) under 808 nm pump light excitation, and it also exhibited satisfactory gain performance at 1.55 μm (*G*_max_ of 6.17 cm^−1^). Consequently, the Er^3+^-doped TeO_2_-Bi_2_O_3_-B_2_O_3_-ZnO glass prepared in this study has the potential for significant applications in near-infrared materials.

## Figures and Tables

**Table 1:** Judd–Ofelt strength parameters for erbium ions in the studied tellurite glass.

Glass	Ω_2_ (×10^−20^ cm^2^)	Ω_4_ (×10^−20^ cm^2^)	Ω_6_ (×10^−20^ cm^2^)	Ref.
TBBZ1	4.31	1.44	1.40	This work
TBBZ2	3.54	1.19	1.14	This work
TBBZ3	3.13	1.05	1.0	This work
TBBZ4	2.80	0.94	0.9	This work
TBBZ5	2.57	0.86	0.82	This work
HYTC0.6	2.84	1.56	1.82	[33]
Tellurite	1.88	1.98	1.39	[34]
Fluoride	3.08	1.46	1.69	[35]

**Table 2:** Calculated spontaneous transition probability (A_rad_), total spontaneous transition probability (∑A), branching ratios (β), and radiative lifetime (τ_rad_) radiative properties of TBBZ3 glass sample for various selected excited states of Er^3+^.

Transition	λ (nm)	A_rad_ (s^−1^)	∑A (s^−1^)	β (%)	τ_rad_ (ms)
^4^I_13/2_→^4^I_15/2_	1533	284.41	284.41	100.00	3.52
^4^I_11/2_→^4^I_13/2_	2678	24.61	612.25	4.02	1.63
→^4^I_15/2_	975	587.65		95.98	
^4^I_9/2_→^4^I_11/2_	4395	1.05	922.91	0.11	1.08
→^4^I_13/2_	1664	65.37		7.08	
→^4^I_15/2_	798	856.49		92.80	

**Table 3:** Micro-parameters of cross-relaxation processes of Er^3+^ in TBBZ3 glass.

Glass	ET	Phonon Number (N) and Contribution Radio (%)		Transfer Constant (10^−40^ cm^6^/s)
TBBZE3	CR	0	1	C_D-A_ = 13.8
		99.999	0.001	

## Data Availability

Data are contained within the article.
